# Radicular Dens Invaginatus: Report of a Rare Case

**DOI:** 10.1155/2012/871937

**Published:** 2012-07-31

**Authors:** V. T. Beena, R. Sivakumar, R. Heera, R. Rajeev, Kanaram Choudhary, Swagatika Panda

**Affiliations:** ^1^Department of Oral Pathology & Microbiology, Government Dental College, Trivandrum, Kerala, Thiruvananthapuram 695011, India; ^2^Department of Oral Pathology & Microbiology, Government Dental College, Kottayam, Kerala, India; ^3^Institute of Dental Sciences, Bhubaneshwar, India

## Abstract

Dens invaginatus is a developmental anomaly resulting from invagination of a portion of crown forming within the enamel organ during odontogenesis. The invagination ranges from a slight pitting (coronal type) to an anomaly occupying most of the crown and root (radicular type). Although a clinical examination may reveal a deep fissure or pit on the surface of an anterior tooth, radiographic examination is the most realistic way to diagnose the invagination. The objective of this case presentation is to report a rare case of radicular dens in dente, which is a rare dental anomaly.

## 1. Introduction

Dens invaginatus is a developmental anomaly resulting from invagination of a portion of crown (enamel organ) during odontogenesis [1]. The invagination ranges from a slight pitting (coronal type) to an anomaly occupying most of the crown and root (radicular type) [2]. While the coronal type of invagination is lined with enamel, the radicular type of invagination is lined with cementum [3, 4]. A clinical examination reveals a deep fissure or pit on the lingual surface of an anterior teeth and an occlusal pit on the posterior teeth. Radiographic examination is the most realistic way to diagnose such anomalies as dense invaginatus [1, 2].

The most popular system used to classify Dens invaginatus given by Oehlers [5]. Invaginations are classified as follows:

- Type 1: invagination ends as a blind sac within the crown.
- Type 2: The invagination extends apically beyond the cemento-enamel junction.
- Type 3: The invagination extends beyond the cemento-enamel junction, and a second “apical foramen” is evident [1, 2, 6].

The objective of this case presentation is to report a rare case of radicular dens in dente. Radicular dens invaginatus is a rare dental anomaly [1, 6].

## 2. Case Report

**Figure 1:**
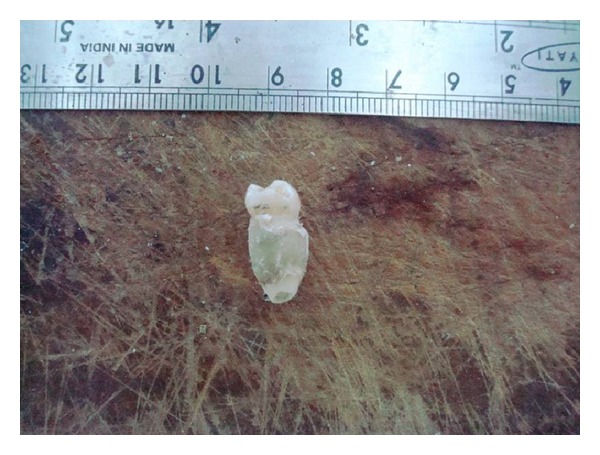
Gross Specimen showing dilation of root.

A 20-year old female presented with a chief complaint of spontaneous, severe and a nocturnal pain in her mandibular right posterior teeth. There was no significant medical history. Extraoral examination revealed no abnormalities. Intraoral examination revealed slight cuspal anomaly in the mandibular right second premolar. This tooth had 3 cups, a small buccal cusp, a small mesiolingual, and a large distolingual cusp but retaining the Y-shaped groove (Figure 1). The tooth was sensitive to vertical and horizontal percussion. There was also horizontal mobility and depressibility. The adjacent gingiva was normal. Extraction of right lower first molar was done before 2 months due to periapical involvement from caries.

**Figure 2:**
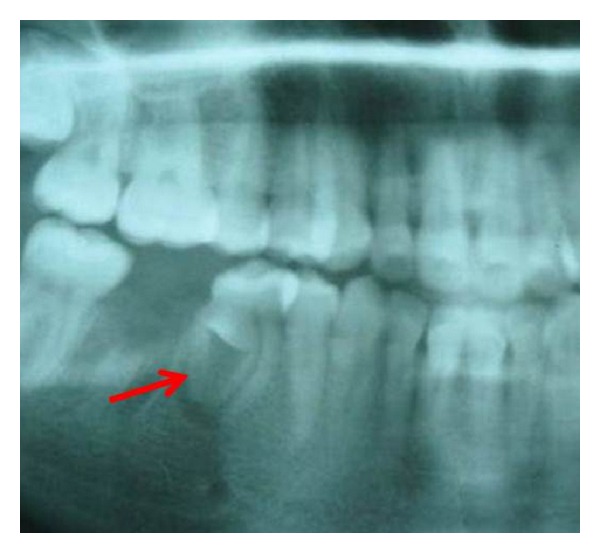
Cropped orthopatomogram showing invagination in root of mandibular right first premolar.

**Figure 3:**
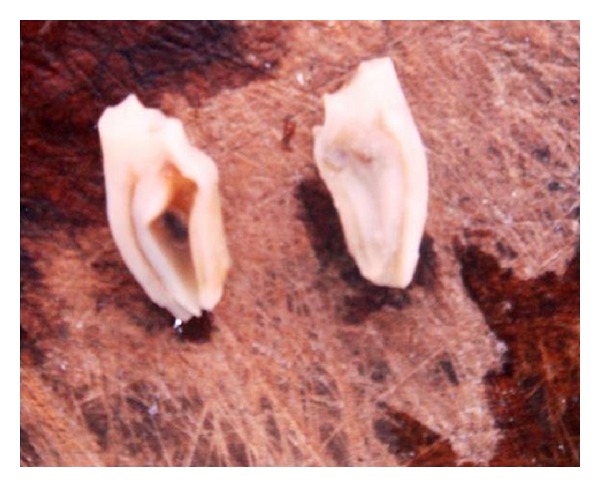
Longitudinal section of involved tooth showing invagination in root of mandibular right first premolar.

The panoramic radiographic examination showed the normal complement of mandibular teeth. The mandibular right 2nd premolar revealed an invagination into the pulpal chamber of the tooth from the radicular portion and periapical radiolucency with an ill-defined border was present around the apex on lateral aspect of the root (Figure 2). The tooth was classified as radicular dens in dente. The pulp extended from the apical foramen to the cervical region of the tooth then split just below the cemento-enamel junction (Figure 3). Because of difficulties in accessing the canals, the tooth was extracted. After the removal of the tooth, the gross findings were noted down. The radicular portion was completely covered circumferentially by a mineralized structure which is yellowish in colour resembling cementum.

## 3. Discussion

Dens invaginatus occurs rarely in the primary teeth but frequently in the permanent dentition and has a general prevalence of 0.04–10% [3, 7, 8]. The more severe forms, however are less common. There is a 3 : 1 female predilection [3, 9]. Dens invaginatus commonly affects the maxillary lateral incisors but occurrence in the mandible is extremely rare. To date approximately 12 cases have been reported. Bilateral occurrence is a typical scenario, but in the present case only the mandibular right lower premolar was involved. According to the Oehlers system [5], the tooth was classified as a Type 2 Dens invaginatus. Bhatt and Dholakia [2] claimed that the radicular invagination usually results from an enfolding of Hertwig's root sheath and originates within the root after development is complete. The dens invaginatus usually presents a bizarre radiographic appearance. The present case depicts the morphologically and anatomically altered tooth structure.

 In Dens invaginatus the invagination area is separated from the pulpal tissues with a thin layer of dentin and frequently communicates with the oral cavity. This allows the entry of irritants and microorganisms, which usually leads to infection and necrosis of the pulpal tissue and may lead to a periodontal or periapical abscess with continuous ingress of irritants. Treatment ranges from restorative procedures, nonsurgical root canal therapy, or extraction [10].

## 4. Summary

Dens invaginatus is clinically significant due to the possibility of pulpal involvement and pulpitis. Necrotic pulps and chronic periapical lesions are often associated with this anomaly without clinical symptoms. Clinicians should be careful of the possibility of Dens invaginatus when a tooth presents pulpitis without a history of trauma or caries and examine the suspicious tooth and periodontium radiographically.
